# Efficacy and safety of Tislelizumab combined with Axitinib as first-line treatment for intermediate- and high-risk metastatic clear-cell renal cell carcinoma

**DOI:** 10.3389/fphar.2025.1618898

**Published:** 2025-05-27

**Authors:** Jie Cui, Pengyong Xu, Changying Guo, Yong Guan, Kejia Zhu, Sentai Ding

**Affiliations:** ^1^ Department of Urology, Yantai Penglai People’s Hospital, Yantai, China; ^2^ Department of Urology, Shandong Provincial Hospital Affiliated to Shandong First Medical University, Jinan, China

**Keywords:** clear-cell renal cell carcinoma, tislelizumab, axitinib, first-line treatment, immunotherapy

## Abstract

**Objective:**

To evaluate the efficacy and safety of Tislelizumab combined with Axitinib in the treatment of intermediate-high risk metastatic clear-cell renal cell carcinoma (ccRCC).

**Methods:**

From September 2021 to June 2023, a total of 20 untreated patients with intermediate-high risk metastatic advanced ccRCC from Shandong Provincial Hospital were included in the study. Clinical characteristics and efficacy were analyzed, and adverse events (AEs) were summarized. All patients received Tislelizumab (200 mg every 3 weeks) and Axitinib (5 mg twice daily bid) until disease progression or intolerable toxicity occurred. The primary endpoint was objective response rate (ORR), and secondary endpoints included disease control rate (DCR), progression-free survival (PFS), overall survival (OS), and incidence of adverse reactions (AEs).

**Results:**

The median follow-up time was 19.0 months (range, 9.2–24.4 months), and the median treatment cycle was 16 (range, 2–16). Partial response was observed in 14 patients (70%), stable disease in 2 patients (10%), and disease progression in 4 patient (20%). The overall ORR was 70.0%, and the DCR was 80.0%. The 1-year OS rate was 100%. The incidence of any grade AEs was 85% (17/20), and the incidence of grade 3–4 AEs was 15% (3/20). Common AEs included gastrointestinal reactions (60%, 12/20), rash (40%, 8/20), and hypertension (30%, 6/20).

**Conclusion:**

Tislelizumab combined with Axitinib as first-line treatment for intermediate-high risk metastatic ccRCC showed significant efficacy and manageable safety.

## 1 Introduction

Renal cell carcinoma (RCC) is one of the most common malignancies of the urinary system, accounting for approximately 3%–5% of newly diagnosed cancer cases (Capitanio et al., 2019). Surgery is the primary treatment for early-stage RCC; however, due to its insidious symptoms, 20%–30% of patients present with distant metastases at the time of initial diagnosis, rendering them ineligible for surgical intervention. Additionally, about 30%–40% of patients who undergo radical surgery will experience a recurrence of metastatic disease (Bahadoram et al., 2022). Once metastasis occurs, the 5-year survival rate for these patients is approximately 10% (Siegel et al., 2022). Although first-line targeted therapies that inhibit TKI(Tyrosine Kinase Inhibitors),including epidermal growth factor receptor (EGFR),vascular endothelial growth factor receptors (VEGFRs),platelet-derived growth factor receptor (PDGFR), fibroblast growth factor receptor (FGFR), KIT,etc., such as sunitinib, axitinib, and pazopanib, have significantly improved the prognosis of patients with advanced RCC, most patients who receive long-term monotherapy with TKI and then followed by VEGFR inhibitors axitinib or Everolimus etc., and eventually develop resistance, failing to achieve a sustained clinical response (Bergers and Hanahan, 2008; Padala et al., 2020).

Multiple studies have shown that adding anti-programmed death receptor-1 (PD-1)/ programmed death receptor ligand 1 (PD-L1) monoclonal antibodies to VEGFR inhibitors exhibits synergistic antitumor activity, significantly improving the efficacy of first-line treatment for advanced RCC (Motzer et al., 2019; Rini et al., 2019a; Powles et al., 2020). Large clinical studies have demonstrated that the objective response rate (ORR) of pembrolizumab combined with axitinib as first-line treatment for advanced RCC is 59.3%, with a progression-free survival (PFS) of 15.4 months (Powles et al., 2020). Consequently, the National Comprehensive Cancer Network (NCCN), the European Association of Urology (EAU), and the Chinese Society of Clinical Oncology (CSCO) guidelines for the diagnosis and treatment of renal cancer have recommended immunotherapy combined with axitinib as a first-line treatment option for advanced RCC. Besides pembrolizumab, clinical studies of other PD-1/PD-L1 inhibitors (such as avelumab, toripalimab, etc.) combined with axitinib have also further validated the efficacy of these targeted immunotherapy combinations (Yan et al., 2024; Choueiri et al., 2025). However, the options for first-line treatment of advanced metastatic RCC with immunotherapy combined with axitinib remain limited.

Tislelizumab is a humanized anti-PD-1 IgG4 monoclonal antibody with a modified Fc region designed to minimize binding to macrophage Fcγ receptors (FcγR). This modification effectively avoids antibody-dependent cellular phagocytosis (ADCP), a potential mechanism for T-cell clearance and resistance to anti-PD-1 antibodies. Additionally, Tislelizumab exhibits a different binding mode compared to other immune checkpoint inhibitors, resulting in superior affinity—approximately 100 times higher than Pembrolizumab and 50 times higher than Nivolumab (Feng et al., 2019; Lee and Keam, 2020; Lee et al., 2020). In 2020, Tislelizumab was approved by the National Medical Products Administration (NMPA) for the treatment of advanced urothelial carcinoma, making it the first immune checkpoint inhibitor approved for a urological cancer indication in China (Ding et al., 2025). Previous studies have shown that the combination of Axitinib and Tislelizumab significantly improves efficacy compared to Axitinib monotherapy (ORR, 59.1% vs. 40.7%; DCR, 81.8% vs. 66.7%) (Wang et al., 2022). Another study investigating the efficacy of Axitinib combined with Tislelizumab in advanced RCC reported an ORR of 50%, with one patient achieving complete response (CR) (Wu C et al., 2021). Therefore, this real-world observational study aims to analyze the efficacy and safety of Tislelizumab combined with Axitinib as first-line treatment in patients with metastatic RCC treated at Shandong Provincial Hospital.

## 2 Materials and methods

### 2.1 Data collection

This study included 20 patients with intermediate-high risk metastatic advanced ccRCC from Shandong Provincial Hospital between September 2021 and June 2023.

### 2.2 Inclusion criteria

(1)Histopathologically confirmed recurrent or metastatic advanced clear cell renal carcinoma. (2) Patients aged 18–75 years. (3) No prior treatment with targeted therapy or immune checkpoint inhibitors. (4) At least one measurable target lesion at enrollment according to RECIST Version 1.1. (5) All acute toxicities from previous anti-tumor treatments resolved to grade 0–1 or to the levels specified in the inclusion/exclusion criteria (except for alopecia and other toxicities deemed not to pose a safety risk by the investigator) according to NCI CTCAE Version 5.0. (6) Expected survival time ≥12 weeks. (7) Karnofsky Performance Status (KPS) score >60, Eastern Cooperative Oncology Group (ECOG) performance status score of 0–2. (8) Adequate organ function, including absolute neutrophil count ≥1.5 × 10^9/L, platelets ≥80 × 10^9/L, hemoglobin ≥9.0 g/dL; total bilirubin ≤1.5×upper limit of normal (ULN), alanine aminotransferase (ALT) and aspartate aminotransferase (AST) ≤2.5×ULN (≤5×ULN for patients with liver metastases); serum creatinine ≤1.25×ULN or creatinine clearance rate ≥60 mL/min.

### 2.3 Treatment protocol

All patients received intravenous Tislelizumab 200 mg every 3 weeks and oral Axitinib (5 mg orally twice daily) until disease progression or intolerable toxicity occurred. The primary endpoint was objective response rate (ORR), and secondary endpoints included disease control rate (DCR), progression-free survival (PFS), overall survival (OS), and incidence of adverse events (AEs).

### 2.4 Efficacy and adverse event evaluation

Efficacy was evaluated using CT or MRI imaging before treatment and every 6 weeks after the start of treatment, according to RECIST Version 1.1. Adverse events were graded according to NCI CTCAE Version 5.0.

### 2.5 Statistical analysis

Statistical analyses were performed using SPSS 16.0 software. Categorical data were expressed as numbers or percentages. Continuous data (e.g., age) were expressed as median (range). The correlation between gene mutations and PD-L1 status with treatment efficacy was analyzed using Fisher’s exact test. Survival curves were generated using the Kaplan-Meier method, and survival times were expressed as median with 95% confidence intervals (CI). A two-sided P-value ≤0.05 was considered statistically significant.

## 3 Results

### 3.1 Clinical data

A total of 20 patients were included in this study, comprising 16 males (80%) and 4 females (20%), with a median age of 60.2 years (range, 33.5–82.3 years). Among them, 14 patients (70%) had an ECOG performance status score of 0–1, and 6 patients (30%) had a score of 2. According to IMDC risk stratification, 15 patients (75%) were classified as intermediate risk, and 5 patients (25%) as high risk. Tumor staging revealed 7 patients (35%) with stage III and 13 patients (65%) with stage IV disease. The most common sites of metastasis were the lungs (9 patients, 45%), bones (5 patients, 25%), lymph nodes (5 patients, 25%), and other sites (4 patients, including the pancreas, omentum, liver and brain). The baseline characteristics of all patients are shown in Table 1.

**TABLE 1:** Baseline characteristics.

Characteristic	patients
Age,Median (range) — yr	60.2 (33.5–82.3)
Male sex — no. (%)	16 (80.0)
ECOG,no. (%)	
0–1	14 (70.0)
2	6 (30.0)
IMDC prognostic risk, no. (%)	
Intermediate	15 (75.0)
Poor	5 (25.0)
Stage, no. (%)	
III	7 (35.0)
IV	13 (65.0)
Sites of metastasis — no. (%)	
Lung	9 (45.0)
Bone	5 (25.0)
Lymph node	5 (25.0)
pancreas	1 (5.0)
omentum	1 (5.0)
brain	1 (5.0)
Liver	1 (5.0)

ECOG, eastern cooperative oncology group; IMDC, international metastatic renal cell carcinoma database consortium.

### 3.2 Efficacy analysis

**FIGURE 1:**
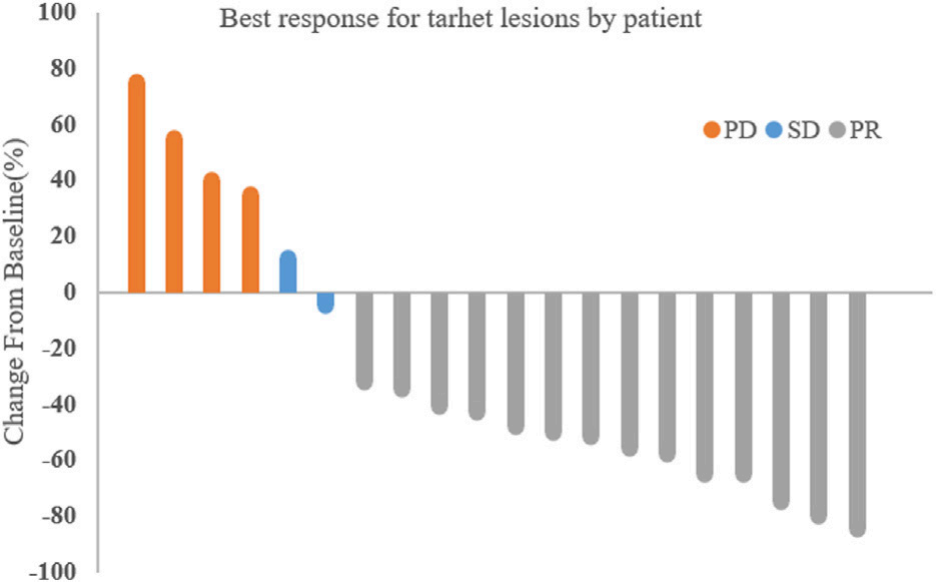
Best response for target lesions by patient.

The median treatment cycle was 16 cycles (range, 2–16), and the median follow-up time was 19.0 months (range, 9.2–24.4 months). All 20 patients were evaluable for efficacy (Figure 1). Among them, 14 patients (70%) achieved partial response (PR), 2 patients (10%) had stable disease (SD), and 4 patients (20%) experienced disease progression (PD). The overall objective response rate (ORR) was 70% (95% CI, 48.9%–84.8%), and the disease control rate (DCR) was 80% (95% CI, 58.4%–91.9%).

### 3.3 Gene mutations and PD-L1 status

**FIGURE 2:**
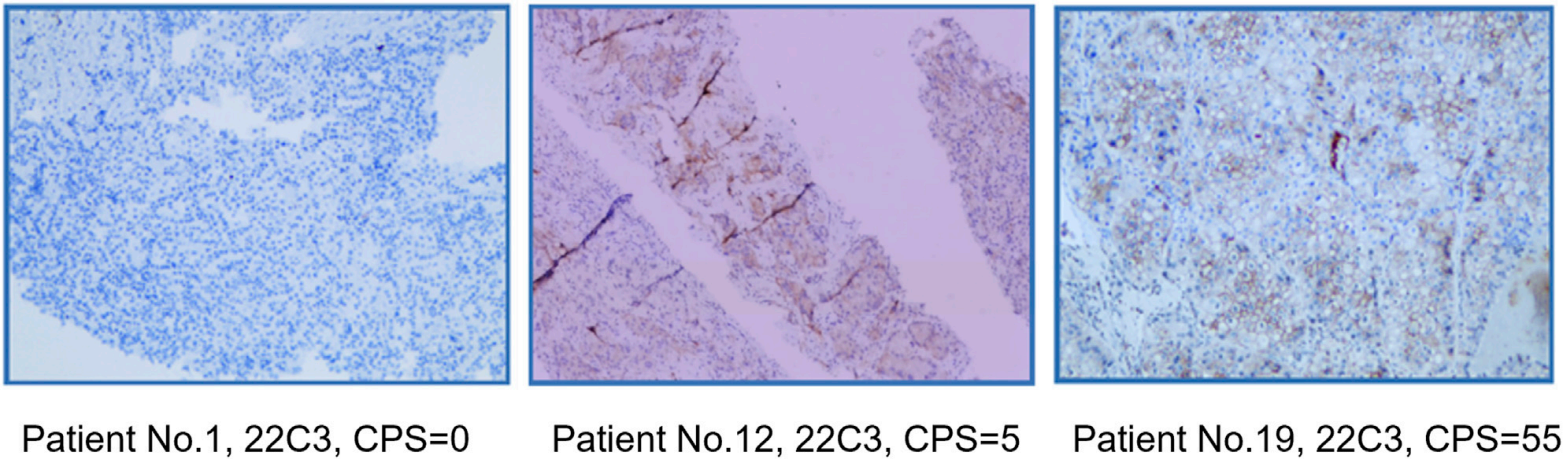
PD-L1 staining image of PD-L1 protein expression level detection.

Using capture-based NGS sequencing (Illumina Hiseq4000), the most common mutations identified in the patients of this study were VHL (40%), BAP1 (25%), and TP53 (25%). The current data did not reveal a significant correlation between VHL mutation status and ORR (p = 0.37). Immunohistochemical analysis was conducted utilizing the 22C3 antibody, where a Combined Positive Score (CPS) greater than 1 was used as the threshold to determine PD-L1 positivity. In this study, the PD-L1 positivity rate was 40% (8/20). Fisher’s exact test analysis showed no correlation between PD-L1 status and ORR (p = 0.67). The PD-L1 and gene mutation status of all patients are shown in Table 2. PD-L1 staining image of PD-L1 protein expression level detection are shown in Figure 2.

**TABLE 2:** PD-L1 and gene mutation status.

No.	PD-L1 (+/−); CPS	Gene mutations
1	(−); 0	VHL S80R、BAP1
2	(+); 5	ARID1A c.4101 + 1G>A、VHL W117S、MET、BAP1
3	(−); 0	MAP2K2 F57L、NONO-TFE3 (N3::T7)、BAP1
4	(+); 60	CDKN2A A36fs、TP53 P75fs、TP53 V157F、BAP1
5	(−); 0	BAP1 S482fs
6	(−); 0	PTEN PIK3CA
7	(+); 20	VHL CHEK1 TP53
8	(−); 0	POLD1、TP53
9	(+); 20	FGFR1、ARID1A
10	(−); 0	VHL H115N
11	(−); 0	MTOR R2368Q
12	(+); 5	TP53 G266R
13	(−); 0	VHL E55Vfs*77
14	(−); 0.1	PIK3CA E707K、TSC1 R37C
15	(−); 0	PIK3CA H1047R、KRAS G12V、TP53 R273C、VHL V130Lfs*29
16	(−); 0	MTOR S2215F、FGFR3 E157K、ATRX D1916N
17	(−); 0	TSC1 R509*、VHL F76Sfs*83
18	(+); 8	TP53 H179R、VHL C162Afs*8
19	(+); 55	ATM Q1531*
20	(+); 1	CCND1 E280V、VHL I206Nfs*50

PD-L1, programmed death receptor ligand 1; CPS, Combined Positive Score; +:PD-L1, positive; -:PD-L1, negative.

### 3.4 Adverse events

Among the 20 patients, the incidence of any grade adverse events (AEs) was 85% (17/20). Most AEs were grade 1–2, with common AEs including diarrhea (60%, 12/20), rash (40%, 8/20), pruritus (30%, 6/20), hypertension (30%, 6/20), and decreased appetite (15%, 3/20). The incidence of grade ≥3 AEs was 15% (3/20). Specifically, One patient was classified as having grade 3 hypertension and required two antihypertensive medications to maintain normal blood pressure levels. Another patient experienced grade 3 skin adverse reactions, leading to permanent discontinuation of the medication and subsequent treatment at a dermatology hospital. A third patient developed grade 3 liver function impairment and grade 3 thrombocytopenia 5 days after the first administration of Tislelizumab. The patient’s aspartate aminotransferase levels peaked at 288 U/L, and platelet count dropped to a minimum of 46 x 10^9/L, resulting in disseminated intravascular coagulation. The patient was admitted to the intensive care unit for treatment, including methylprednisolone pulse therapy. Three days later, the adverse reactions reduced to grade 1. It was decided to suspend Tislelizumab and continue with Pazopanib targeted therapy.

## 4 Discussion

This study is the first report on the efficacy and safety of Tislelizumab combined with Axitinib as first-line treatment for Chinese patients with intermediate-high risk metastatic RCC. The results showed that the 20 patients had favorable tumor response and safety profiles, with an ORR of 70%, a DCR of 80%.

Compared to previous trials involving unselected patient populations, this real-world study focused on a higher-risk population, with all enrolled patients being classified as intermediate-high risk according to IMDC stratification (100% vs. 68%–80%) (Motzer et al., 2018; Motzer et al., 2019; Rini et al., 2019a; Rini et al., 2019b; Choueiri et al., 2021; Motzer et al., 2021; Yan et al., 2024). In this study, treatment-naive metastatic RCC patients receiving the combination of Axitinib and Tislelizumab demonstrated significantly higher ORR values compared to most similar treatment regimens (70% vs. 37.0%–59.3%) (Motzer et al., 2019; Rini et al., 2019a; Yan et al., 2024), confirming its efficacy as a first-line treatment for metastatic RCC. Additionally, due to the short follow-up period, the PFS and OS data are not yet mature. Despite the significant efficacy observed, the results of cross-comparisons should be interpreted with caution; furthermore, due to the small sample size and study design limitations, larger phase III trials are needed to validate the efficacy of this combination therapy.

In terms of safety, Tislelizumab combined with Axitinib was well-tolerated. The incidence of any grade AEs in this study was 94.1%, with common events such as gastrointestinal reactions, skin reactions, and hypertension, which were generally tolerable and manageable, resolving quickly after symptomatic treatment. Additionally, the incidence of grade ≥3 AEs was 17.6%, including hypertension, skin reactions, and liver toxicity, which were successfully controlled through necessary dose reductions or treatment interruptions. Hypertension was one of the most common AEs in this study, as well as a typical treatment-related toxicity in other immunotherapy combined with Axitinib regimens. Compared to safety data reported abroad, the incidence of any grade (35.3% vs. 49.5%) and grade ≥3 (5.9% vs. 25.6%) hypertension in this study was lower (Rini et al., 2019a). Overall, Tislelizumab combined with Axitinib was well-tolerated by most patients.

In this study, no significant correlation was observed between PD-L1 positivity (40%) and ORR (p = 0.67). Another RENOTORCH study of axitinib in combination with teraplizumab included patients with intermediate- to high-risk advanced renal cancer, with an ORR of 58.54% (24/41) for the combination in 24 high-risk patients assessed by an independent review committee, but no data on PD-L1 status and efficacy (Yan et al., 2024). The synergistic effect of axitinib with immunotherapy has been demonstrated to enhance T-cell infiltration in tumours, thereby partially counteracting immunosuppression in PD-L1-negative patients (Atkins MB, et al., 2018). Kidney cancer develops in 25%–45% of VHL patients and is uniformly clear cell, bilateral, and multifocal (Schmidt and Linehan, 2016). In this study, the VHL mutation rate was found to be 40% in the cohort of high-risk patients. No significant association was identified between VHL mutation status and objective response rate (p = 0.37). Furthermore, it was hypothesised that genomic complexity (e.g., chromosomal copy number variation, epigenetic remodelling) in high-risk populations may mask the predictive value of a single gene (e.g., VHL). It is noteworthy that, although VHL mutation status was not significantly associated with ORR (p = 0.37), BAP1/TP53 co-mutations (10% of this cohort) may be associated with poorer treatment response.

This study also has some limitations: (1) The follow-up period was short, and survival indicators are not yet complete, requiring further exploration of the long-term survival outcomes and safety of Tislelizumab combined with Axitinib; (2) Due to the study design and sample size limitations, all interpretations of the results are preliminary, and larger trials are needed in the future to further validate the clinical practice value of this combination regimen.

In summary, this real-world prospective study demonstrates that Tislelizumab combined with Axitinib as first-line treatment for intermediate-high risk metastatic RCC is effective and safe. This study suggests that this combination regimen is a feasible treatment strategy for intermediate-high risk metastatic RCC.

## Data Availability

The original contributions presented in the study are included in the article/supplementary material, further inquiries can be directed to the corresponding author.

## References

[B1] Atkins M. B. Plimack E. R. Puzanov I. Fishman M. N. McDermott D. F. Cho D. C. (2018). Axitinib in combination with pembrolizumab in patients with advanced renal cell cancer: a non-randomised, open-label, dose-finding, and dose-expansion phase 1b trial. Lancet Oncol. 19, 405–415. 10.1016/S1470-2045(18)30081-0 29439857 PMC6860026

[B2] Bahadoram S. Davoodi M. Hassanzadeh S. Bahadoram M. Barahman M. Mafakher L. (2022). Renal cell carcinoma: an overview of the epidemiology, diagnosis, and treatment. G. Ital. Nefrol. 39 (3), 2022. 35819037

[B3] Bergers G. Hanahan D. (2008). Modes of resistance to anti-angiogenic therapy. Nat. Rev. Cancer 8 (8), 592–603. 10.1038/nrc2442 18650835 PMC2874834

[B4] Capitanio U. Bensalah K. Bex A. Boorjian S. A. Bray F. Coleman J. (2019). Epidemiology of renal cell carcinoma. Eur. Urol. 75 (1), 74–84. 10.1016/j.eururo.2018.08.036 30243799 PMC8397918

[B5] Choueiri T. K. Penkov K. Uemura H. Campbell M. T. Pal S. Kollmannsberger C. (2025). Avelumab + axitinib versus sunitinib as first-line treatment for patients with advanced renal cell carcinoma: final analysis of the phase III JAVELIN Renal 101 trial. Ann. Oncol. 36 (4), 387–392. 10.1016/j.annonc.2024.12.008 39706335 PMC12184433

[B6] Choueiri T. K. Powles T. Burotto M. Escudier B. Bourlon M. T. Zurawski B. (2021). Nivolumab plus Cabozantinib versus sunitinib for advanced renal-cell carcinoma. N. Engl. J. Med. 384 (9), 829–841. 10.1056/NEJMoa2026982 33657295 PMC8436591

[B7] Ding S. Wu C. Cao J. Lyu J. (2025). Immune checkpoint inhibitor therapy as a neoadjuvant treatment for muscle-invasive bladder carcinoma: a narrative review. Curr. Urol. 19 (1), 39–42. 10.1097/cu9.0000000000000263 40313419 PMC12042169

[B8] Feng Y. Hong Y. Sun H. Zhang B. Wu H. Li K. (2019). Abstract 2383: the molecular binding mechanism of tislelizumab, an investigational anti-PD-1 antibody, is differentiated from pembrolizumab and nivolumab. Cancer Res. 79 (13_Suppl. ment), 2383. 10.1158/1538-7445.am2019-2383

[B9] Lee A. Keam S. J. (2020). Tislelizumab: first approval. Drugs 80 (6), 617–624. 10.1007/s40265-020-01286-z 32185681

[B10] Lee S. H. Lee H. T. Lim H. Kim Y. Park U. B. Heo Y. S. (2020). Crystal structure of PD-1 in complex with an antibody-drug tislelizumab used in tumor immune checkpoint therapy. Biochem. Biophys. Res. Commun. 527 (1), 226–231. 10.1016/j.bbrc.2020.04.121 32446372

[B11] Motzer R. Alekseev B. Rha S. Y. Porta C. Eto M. Powles T. (2021). Lenvatinib plus pembrolizumab or Everolimus for advanced renal cell carcinoma. N. Engl. J. Med. 384 (14), 1289–1300. 10.1056/NEJMoa2035716 33616314

[B12] Motzer R. J. Penkov K. Haanen J. Rini B. Albiges L. Campbell M. T. (2019). Avelumab plus axitinib versus sunitinib for advanced renal-cell carcinoma. N. Engl. J. Med. 380 (12), 1103–1115. 10.1056/NEJMoa1816047 30779531 PMC6716603

[B13] Motzer R. J. Tannir N. M. McDermott D. F. Aren Frontera O. Melichar B. Choueiri T. K. (2018). Nivolumab plus Ipilimumab versus sunitinib in advanced renal-cell carcinoma. N. Engl. J. Med. 378 (14), 1277–1290. 10.1056/NEJMoa1712126 29562145 PMC5972549

[B14] Padala S. A. Barsouk A. Thandra K. C. Saginala K. Mohammed A. Vakiti A. (2020). Epidemiology of renal cell carcinoma. World J. Oncol. 11 (3), 79–87. 10.14740/wjon1279 32494314 PMC7239575

[B15] Powles T. Plimack E. R. Soulieres D. Waddell T. Stus V. Gafanov R. (2020). Pembrolizumab plus axitinib versus sunitinib monotherapy as first-line treatment of advanced renal cell carcinoma (KEYNOTE-426): extended follow-up from a randomised, open-label, phase 3 trial. Lancet Oncol. 21 (12), 1563–1573. 10.1016/S1470-2045(20)30436-8 33284113

[B16] Rini B. I. Plimack E. R. Stus V. Gafanov R. Hawkins R. Nosov D. (2019a). Pembrolizumab plus axitinib versus sunitinib for advanced renal-cell carcinoma. N. Engl. J. Med. 380 (12), 1116–1127. 10.1056/NEJMoa1816714 30779529

[B17] Rini B. I. Powles T. Atkins M. B. Escudier B. McDermott D. F. Suarez C. (2019b). Atezolizumab plus bevacizumab versus sunitinib in patients with previously untreated metastatic renal cell carcinoma (IMmotion151): a multicentre, open-label, phase 3, randomised controlled trial. Lancet 393 (10189), 2404–2415. 10.1016/S0140-6736(19)30723-8 31079938

[B18] Schmidt L. S. Linehan W. M. (2016). Genetic predisposition to kidney cancer. Semin. Oncol. 43, 566–574. 10.1053/j.seminoncol.2016.09.001 27899189 PMC5137802

[B19] Siegel R. L. Miller K. D. Fuchs H. E. Jemal A. (2022). Cancer statistics, 2022. CA Cancer J. Clin. 72 (1), 7–33. 10.3322/caac.21708 35020204

[B20] Wang Z. Chen J. Li Q. Li N. Yu J. Liu F. (2022). The effects of axitinib plus tislelizumab in the treatment of advanced renal cell carcinoma. J. Oncol. 2022, 2700166. 10.1155/2022/2700166 35368892 PMC8970885

[B21] Wu C. Huang Z. Wu B. Zhang W. Chen X. Lv L. (2021). Efficacy of axitinib combined with tislelizumab in patients with advanced renal cell carcinoma who failed first-line targeted therapy. Chin. J. Clin. Pharmacol. 37 (12), 1501–1504.

[B22] Yan X. Q. Ye M. J. Zou Q. Chen P. He Z. S. Wu B. (2024). Toripalimab plus axitinib versus sunitinib as first-line treatment for advanced renal cell carcinoma: RENOTORCH, a randomized, open-label, phase III study. Ann. Oncol. 35 (2), 190–199. 10.1016/j.annonc.2023.09.3108 37872020

